# Durvalumab as monotherapy and in combination therapy in patients with lymphoma or chronic lymphocytic leukemia: The FUSION NHL 001 trial

**DOI:** 10.1002/cnr2.1662

**Published:** 2022-07-19

**Authors:** Carla Casulo, Armando Santoro, Guillaume Cartron, Kiyoshi Ando, Javier Munoz, Steven Le Gouill, Koji Izutsu, Simon Rule, Pieternella Lugtenburg, Jia Ruan, Luca Arcaini, Marie‐Laure Casadebaig, Brian Fox, Nurgul Kilavuz, Nils Rettby, Justine Dell'Aringa, Lilia Taningco, Richard Delarue, Myron Czuczman, Thomas Witzig

**Affiliations:** ^1^ University of Rochester Medical Center Wilmot Cancer Institute Rochester New York USA; ^2^ Humanitas University and Humanitas Clinical and Research Center IRCCS Milan Italy; ^3^ Centre Hospitalier Universitaire de Montpellier Montpellier France; ^4^ Tokai University School of Medicine Isehara Japan; ^5^ Banner MD Anderson Cancer Center Gilbert Arizona USA; ^6^ Service d'hématologie clinique du CHU de Nantes INSERM CRCINA Nantes‐Angers, NeXT Université de Nantes Nantes France; ^7^ National Cancer Center Hospital Tokyo Japan; ^8^ University of Plymouth Plymouth UK; ^9^ University Medical Center Rotterdam Erasmus MC Cancer Institute Rotterdam The Netherlands; ^10^ Weill Cornell Medicine New York New York USA; ^11^ Division of Hematology, Fondazione IRCSS Policlinico San Matteo and Department of Molecular Medicine University of Pavia Pavia Italy; ^12^ Bristol Myers Squibb Princeton New Jersey USA; ^13^ Mayo Clinic Rochester Minnesota USA

**Keywords:** clinical trials, durvalumab, hematologic malignancies, lymphoma, programmed death ligand 1

## Abstract

**Background:**

Studies suggest that immune checkpoint inhibitors may represent a promising strategy for boosting immune responses and improving the antitumor activity of standard therapies in patients with relapsed/refractory hematologic malignancies.

**Aims:**

Phase 1/2 FUSION NHL 001 was designed to determine the safety and efficacy of durvalumab, an anti‐programmed death ligand 1 (PD‐L1) antibody, combined with standard‐of‐care therapies for lymphoma or chronic lymphocytic leukemia (CLL).

**Methods and Results:**

The primary endpoints were to determine the recommended phase 2 dose of the drugs used in combination with durvalumab (durvalumab was administered at the previously recommended dose of 1500 mg every 4 weeks) and to assess safety and tolerability. Patients were enrolled into one of four arms: durvalumab monotherapy (Arm D) or durvalumab in combination with lenalidomide ± rituximab (Arm A), ibrutinib (Arm B), or rituximab ± bendamustine (Arm C). A total of 106 patients with relapsed/refractory lymphoma were enrolled. All but two patients experienced at least one treatment‐emergent adverse event (TEAE); those not experiencing a TEAE were in Arm C (diffuse large B‐cell lymphoma [DLBCL]) and Arm D (DLBCL during the durvalumab monotherapy treatment period). No new safety signals were identified, and TEAEs were consistent with the respective safety profiles for each study treatment. Across the study, patients with follicular lymphoma (FL; *n* = 23) had an overall response rate (ORR) of 59%; ORR among DLBCL patients (*n* = 37) was 18%. Exploratory biomarker analysis showed that response to durvalumab monotherapy or combination therapy was associated with higher interferon‐γ signature scores in patients with FL (*p* = .02).

**Conclusion:**

Durvalumab as monotherapy or in combination is tolerable but requires close monitoring. The high rate of TEAEs during this study may reflect on the difficulty in combining durvalumab with full doses of other agents. Durvalumab alone or in combination appeared to add limited benefit to therapy.

## 1 INTRODUCTION

Accumulating evidence suggests that therapies targeting immune cells in the tumor microenvironment have the capacity to improve the antitumor activity of standard therapies in patients with relapsed/refractory hematologic malignancies.^1^, ^2^, ^3^ Immunomodulatory drugs such as lenalidomide and anti‐programmed cell death‐1 (PD‐1)/programmed death ligand 1 (PD‐L1) therapeutics including nivolumab have shown promise as monotherapy for chemorefractory Hodgkin and non‐Hodgkin lymphoma.^1^, ^4^ Clinical data also suggest that anti‐PD‐1/PD‐L1 therapeutics, such as durvalumab, may improve the activity of lymphoma therapies including lenalidomide (an immunomodulatory drug), ibrutinib (a Bruton's tyrosine kinase inhibitor), bendamustine (an alkylating pro‐apoptotic chemotherapeutic), and rituximab (a monoclonal anti‐CD20 antibody) when given in combination.^5^, ^6^, ^7^, ^8^ Preclinical evidence in murine lymphoma models suggests synergistic antitumor activity with ibrutinib plus durvalumab.^3^

Several reports have described a combination treatment approach with PD‐L1/PD‐1‐targeted therapy, including ibrutinib and nivolumab in non‐Hodgkin lymphoma (NHL)^9^ and chronic lymphocytic leukemia (CLL) and lenalidomide with pembrolizumab in double‐hit lymphoma.^10^ The use of immune checkpoint inhibitors (ICIs) is also supported by the increased expression of PD‐L1/PD‐1 in tumor‐infiltrating lymphocytes from patients with lymphoma and increased PD‐1 expression in circulating T cells from patients with CLL.^11^ Studies also showed benefits of using nivolumab or pembrolizumab in relapsed/refractory classical Hodgkin lymphoma (HL)^1^, ^12^; pidilizumab with rituximab in follicular lymphoma (FL)^13^; and nivolumab in a small percentage of patients with diffuse large B‐cell lymphoma (DLBCL), FL, or T‐cell lymphomas.^14^ Durvalumab, a monoclonal antibody that binds to PD‐L1 to block its interaction with programmed cell death‐1 PD‐1, is approved in the United States and other countries for the treatment of several nonhematologic cancers.^15^, ^16^ Based on initial studies that suggested potential antitumor activity in hematologic malignancies with anti‐PD‐1/PD‐L1 therapeutics, we hypothesized that the T‐cell‐mediated anti‐tumor responses seen after treatment with durvalumab might act synergistically with standard‐of‐care treatments for lymphoma and CLL, ultimately resulting in enhanced efficacy without compromising safety.^17^, ^18^

The aim of the FUSION NHL 001 study was to determine the safety and tolerability of durvalumab when given in combination with lenalidomide ± rituximab, ibrutinib, or rituximab ± bendamustine in patients with lymphoma or CLL. The safety and efficacy of durvalumab were also assessed in patients receiving the recommended phase 2 dose (RP2D) of these treatment regimens as well as durvalumab monotherapy. Previously reported data showed that PD‐L1 expression is associated with poor prognosis in patients with DLBCL.^19^ In addition, in patients with non‐small cell lung cancer or urothelial cancer receiving durvalumab monotherapy, elevated PD‐L1 expression and interferon (IFN)‐γ scores in baseline tumor biopsy samples were associated with higher overall reaponse rate (ORR) or improved survival.^20^, ^21^ In the current study, an exploratory biomarker analysis was also performed to assess the expression of PD‐L1, CD8, and an IFN‐signature score signature^20^ and how they associate with response to treatment with durvalumab.

## 2 METHODS

### 2.1 Patients

**FIGURE 1:**
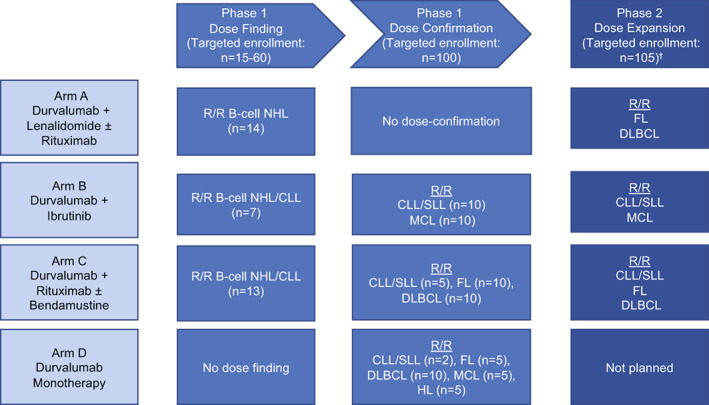
Study design schematic. Numbers represent the number of targeted patients for each phase and the actual number pf patients enrolled for each arm. ^†^Not opened. CLL, chronic lymphocytic leukemia; DLBCL, diffuse large B‐cell lymphoma; FL, follicular lymphoma; HL, Hodgkin lymphoma; MCL, mantle cell lymphoma; NHL, non‐Hodgkin lymphoma; R/R, relapsed/refractory, SLL, small lymphocytic lymphoma.

Key eligibility criteria: patients aged ≥18 to ≤80 years at the time of signing the informed consent form, histologically confirmed and documented eligible histologies (relapsed/refractory B‐cell NHL, CLL, FL, DBLCL, small lymphocytic lymphoma [SLL], mantle cell lymphoma [MCL], HL; see Figure 1 for details) assessed by the investigator and local pathologist per the 2008 World Health Organization Lymphoma Classification^22^; treatment with at least 1 prior systemic chemotherapy, immunotherapy, or chemoimmunotherapy; high‐risk CLL/SLL (defined as the presence of at least one of the following: complex karyotype, del (17p) abnormality, mutated TP53, ibrutinib or other Bruton's tyrosine kinase inhibitor failure, or relapsed/progressive disease within 6 months of completing their last therapy); documented active relapsed or refractory disease requiring therapeutic intervention; Eastern Cooperative Oncology Group (ECOG) performance status 0–2; and life expectancy >6 months. Complete inclusion/exclusion criteria overall and by treatment arm are indicated in Table S1.

### 2.2 Study design and treatments

The trial design is presented in Figure 1. This was a phase 1/2, open‐label, international, multicenter study (NCT02733042) initially planned in three parts with four treatments. Phase 1 consisted of a dose‐finding and a dose‐confirmation part, and phase 2 was a planned dose‐expansion part. Patients were assigned to one of the four treatment arms that evaluated durvalumab in combination with lenalidomide ± rituximab (Arm A), ibrutinib (Arm B), rituximab ± bendamustine (Arm C), or as monotherapy (Arm D). The entire study was anticipated to last approximately 8 years, with an anticipated study duration for any patient of up to approximately 3–8 years depending on the assigned treatment arm and patient's disease histology.

The first patient visit was on May 3, 2016. On September 5, 2017, enrollment of new patients into Arm A was discontinued, based on a partial clinical hold placed by the US Food and Drug Administration resulting from risks identified in trials of pembrolizumab in combination with immunomodulatory agents in patients with multiple myeloma. Patients already enrolled and treated who were receiving clinical benefit from combination treatment could continue combination treatment after being informed and reconsented on the safety concerns with the combination of a PD‐1 pathway inhibitor and an immunomodulatory agent.

The durvalumab dose was fixed in each of the arms. Eligible relapsed/refractory disease histologies were B‐cell NHL (Arm A) and B‐cell NHL and CLL (Arms B and C). The dose‐confirmation part (phase 1; Arms A, B, C, and D) enrolled patients with each prespecified disease histology to confirm the tolerability and safety of these combinations and identify the strongest antitumor signal in those histologies. Eligible relapsed/refractory disease histologies in the dose‐confirmation cohort were FL and DLBCL (Arm A); CLL/SLL and mantle cell lymphoma (MCL; Arm B); CLL/SLL, FL, and DLBCL (Arm C); and CLL/SLL, DLBCL, FL, MCL, and HL (Arm D).

During each 28‐day treatment cycle, durvalumab was administered by intravenous (IV) infusion on day 1 of cycles 1–13 at a fixed dose of 1500 mg every 4 weeks (Q4W) in combination with: lenalidomide 20 or 10 mg (based on pretreatment creatinine clearance) orally once daily (QD) on days 1–21 of each cycle for cycles 1–13 with or without rituximab 375 mg/m^2^ IV infusion (Arm A); ibrutinib 560 mg (NHL) or 420 mg (CLL) orally QD continuously until disease progression (Arm B); rituximab 375 mg/m^2^ IV infusion on day 2 of cycles 1−6 with or without bendamustine 90 mg/m^2^ (NHL) or 70 mg/m^2^ (CLL) IV infusion on days 1 and 2 of cycles 1−6 (Arm C); or durvalumab monotherapy (Arm D). Response to treatment was determined by the Lugano Classification for lymphoma and the International Workshop on Chronic Lymphocytic Leukemia Response Criteria for CLL.^23^, ^24^

The study was conducted in compliance with International Council for Harmonization Good Clinical Practice, and in accordance with the general ethical principles outline in the Declaration of Helsinki and applicable national, state, and local laws of the pertinent regulatory authorities. The protocol was approved by an institutional review board/independent ethics committee prior to commencement.

### 2.3 Endpoints and assessments

#### 2.3.1 Safety

Primary endpoints for the dose‐finding part of the study were to determine the RP2D of each combination therapy (including dose‐limiting toxicities [DLTs]) and to assess the safety and tolerability of durvalumab when given in combination with lenalidomide and rituximab or bendamustine and rituximab. To assess safety, the incidence of treatment‐emergent adverse events (TEAEs) using National Cancer Institute Common Terminology Criteria for Adverse Events version 4.03 was evaluated. During dose confirmation, primary endpoints assessed the safety of durvalumab as monotherapy and when given in combination with lenalidomide and rituximab, ibrutinib, or bendamustine and rituximab by examining the incidence of TEAEs.

#### 2.3.2 Efficacy

During dose‐expansion, the primary preliminary efficacy endpoint was ORR based on tumor‐specific response criteria (e.g., Lugano Classification and International Workshop on Chronic Lymphocytic Leukemia response criteria). Secondary endpoints for dose‐finding and confirmation parts of the study included ORR based on tumor‐specific response criteria, time to response (TTR), duration of response (DoR), and progression‐free survival (PFS). Overall survival was also examined.

### 2.4 Exploratory biomarker analyses

Among patients with lymphoma, biopsies were collected within 28 days before cycle 1 day 1 (mandatory) and any time during cycle 2 (strongly recommended); in some cases, archival formalin‐fixed paraffin‐embedded biopsies were used. Biopsy slides were sent to Q^2^ Solutions/EA Genomics (Durham, NC, USA) and RNA was extracted using the Qiagen micro RNeasy FFPE Kit (Qiagen, Hilden, Germany). Quality and quantity of recovered RNA was determined by both Bioanalyzer (Agilent Technologies, Santa Clara, CA, USA) and Qubit (Thermo Fisher Scientific, Waltham, MA, USA). The DV200 metric (Agilent Technologies) and ribogreen RNA quantitation (Thermo Fisher Scientific) identified the percentage of RNA fragments >200 nucleotides. Following extraction, the TruSeq RNA Exome kit (library prep, RNA enrichment, index adapters; Illumina, San Diego, CA, USA) was used to create barcoded libraries, which were quantified. Each sample was processed using the TruSeq RNA Exome Kit and library material was loaded onto a HiSeq 2500 (Illumina) or equivalent. Alignment was performed using a two‐pass mode with STAR (v2.5.2b) on the full hg38 human genome, DESeQ^25^ was used to normalize counts with the function “CalcNormFactors,” followed by the “cpm” function to generate counts per million (CPM) estimates. An IFN‐γ signature score was calculated by taking the mean CPM of four genes (*CD274* [PD‐L1]; *LAG3*, *CXCL9*, and *IFNG*), as described by Higgs et al.^20^

Immunohistochemistry (IHC) was performed by Geneuity Clinical Research Services (Maryville, TN, USA) using antibodies to CD8 and PD‐L1. CD8 was quantified as the number of positive cells per square millimeter. PD‐L1 was quantified as the number of PD‐L1 positive cells per square millimeter and also a visual estimate of the percent of tumor cells which are positive for PD‐L1. The antibody clones used were clone 4B11 for CD8 and SP142 for PD‐L1.

### 2.5 Statistical analysis

Sample‐size determination established that a maximum of 60 patients was required for the dose‐finding part, and a maximum of 100 patients was required for the dose‐confirmation part. Celgene, together with AstraZeneca/MedImmune, decided that the dose‐expansion part was not to be opened. As a consequence, the entire study was to enroll a maximum of approximately 160 patients. All primary and secondary statistical analyses were conducted with SAS® version 9.1 or higher (SAS Institute, Inc., Cary, North Carolina, USA). The safety population included all patients who received at least one dose of study drug. The efficacy population included all patients who completed at least one cycle of their assigned treatment and had baseline and at least one post‐baseline tumor response assessment. If multiple values were present for the same date, the mean of these values was reported (for character parameters like urinalysis, the worst value was reported). Confidence intervals (CIs) were presented as two‐sided 95% CIs unless specified. For translational analyses, a *t‐*test (using R version 3.6) was used to compare the nonresponders with the responders within each histology group for IFN‐γ signature score. This specific score and corresponding analysis approach were prespecified before generating the gene expression data.

## 3 RESULTS

### 3.1 Patients and treatment

This study was conducted from May 3, 2016, to March 6, 2019, at clinical sites in the United States, France, Italy, the Netherlands, Japan, and the United Kingdom. A total of 106 patients were enrolled. Fourteen patients were enrolled in Arm A; 7 and 20 patients were enrolled in Arm B dose‐finding and dose‐confirmation cohorts, respectively; 13 and 25 patients were enrolled in Arm C dose‐finding and dose‐confirmation cohorts, respectively; and 27 patients were enrolled in Arm D. Demographics and baseline characteristics of the dose‐finding and dose‐confirmation cohorts are summarized in Table 1.

**TABLE 1:** Demographics and baseline characteristics by treatment arm (safety population)

Parameter	Arm A durvalumab combinations			Arm B durvalumab combinations		Arm C durvalumab combinations				Arm D durvalumab monotherapy 1500 mg			
Dose‐finding cohort	Len 20 mg	R^a^ + Len 20 mg	R^a^ + Len 10 mg	Ibr 420 mg	Ibr 560 mg	R^a^	Ben 70 mg	R^a^ + Ben 70 mg	R^a^ + Ben 90 mg	No dose‐finding			
*n*	3	3	8	3	4	3	1	4	5				
Age, median (range), y	71 (50–78)	66 (52–75)	77 (53–80)	58 (54–74)	68 (57–81)	79 (76–80)	70 (70–70)	68 (52–78)	38 (21–77)				
Sex, *n* (%)													
Male	2 (67)	3 (100)	6 (75)	3 (100)	2 (50)	1 (33)	1 (100)	3 (75)	2 (40)				
Female	1 (33)	0	2 (25)	0	2 (50)	2 (67)	0	1 (25)	3 (60)				
Histology, *n*													
CLL/SLL	0	0	0	1	0	0	0	0	0				
FL	1	3	1	1	0	1	0	1	0				
DLBCL	2	0	4	0	0	2	1	3	5				
MZL	0	0	2	0	3	0	0	0	0				
MCL	0	0	0	1	1	0	0	0	0				
tFL	0	0	1	0	0	0	0	0	0				
ECOG performance status, *n* (%)													
0/1	3 (100)	3 (100)	6 (75)	3 (100)	4 (100)	3 (100)	0	3 (75)	4 (80)				
2/3	0	0	2 (25)	0	0	0	1 (100)	1 (25)	1 (20)				
Number of prior systemic regimens, *n* (%)													
1	1 (33)	0	2 (25)	1 (33)	1 (25)	1 (33)	0	0	1 (20)				
2	2 (67)	2 (67)	2 (25)	0	1 (25)	0	1 (100)	1(25)	1 (20)				
3	0	0	1 (13)	1 (33)	0	0	0	1 (25)	2 (40)				
≥4	0	1 (33)	3 (38)	1 (33)	2 (50)	2 (67)	0	2 (50)	1 (20)				
SD/PD as best response to last treatment, *n* (%)	1 (33)	0	3 (38)	0	1 (25)	0	1 (100)	3 (75)	3 (60)				
Ann Arbor stage, *n* (%)													
1/2	0	2 (67)	2 (25)	1 (33)	0	2 (67)	0	2 (50)	0				
3/4	3 (100)	1 (33)	6 (75)	1 (33)	4 (100)	1 (33)	1 (100)	2 (50)	5 (100)				
Missing	0	0	0	1 (33)	0	0	0	0	0				
Bulky disease ≥7 cm, *n* (%)	1 (33)	0	3 (38)	0	0	1 (33)	0	2 (50)	2 (40)				
LDH > ULN, *n* (%)	1 (33)	1 (33)	5 (63)	0	2 (50)	2 (67)	1 (100)	2 (50)	4 (80)				

Dose‐confirmation cohort				Ibr 420 mg	Ibr 560 mg	R^a^ + Ben 70 mg				Durvalumab monotherapy 1500 mg			
				CLL/ SLL	MCL	FL	DLBCL	CLL/ SLL	FL	DLBCL	CLL/ SLL	MCL	HL
*n*				10	10	10	10	5	5	10	2	5	5
Age, median (range), y				68 (55–73)	74 (54–84)	65 (45–75)	60 (46–71)	68 (53–79)	52 (39–71)	62 (22–76)	62 (55–69)	77 (69–80)	51 (34–65)
Sex, *n* (%)													
Male				7 (70)	9 (90)	7 (70)	6 (60)	3 (60)	3 (60)	6 (60)	1 (50)	3 (60)	3 (60)
Female				3 (30)	1 (10)	3 (30)	4 (40)	2 (40)	2 (40)	4 (40)	1 (50)	2 (40)	2 (40)
ECOG performance status, *n* (%)	No dose‐confirmation												
0/1				10 (100)	9 (90)	9 (90)	8 (80)	4 (80)	4 (80)	7 (70)	2 (100)	5 (100)	5 (100)
2/3				0	1 (10)	1 (10)	2 (20)	1 (20)	1 (20)	3 (30)	0	0	0
Number of prior systemic regimens, *n* (%)													
1				5 (50)	7 (70)	6 (60)	0	0	0	0	0	2 (40)	0
2				3 (30)	2 (20)	2 (20)	4 (40)	2 (40)	0	1 (10)	1 (50)	0	0
3				2 (20)	1 (10)	2 (20)	5 (50)	2 (40)	1 (20)	5 (50)	0	1 (20)	0
≥4				0	0	0	1 (10)	1 (20)	4 (80)	4 (40)	1 (50)	2 (40)	5 (100)
SD/PD as best response to last treatment, *n* (%)				3 (30)	2 (20)	1 (10)	6 (60)	1 (20)	5 (100)	6 (60)	2 (100)	0	4 (80)
Bulky disease ≥7 cm, *n* (%)				2 (20)	4 (40)	4 (40)	1 (10)	3 (60)	2 (40)	5 (50)	1 (50)	2 (40)	1 (20)
LDH > ULN, *n* (%)				4 (40)	4 (40)	4 (40)	7 (70)	2 (40)	3 (60)	7 (70)	1 (50)	1 (20)	2 (40)

Abbreviations: Ben, bendamustine; CLL, chronic lymphocytic leukemia; DLBCL, diffuse large B cell lymphoma; ECOG, Eastern Cooperative Oncology Group; FL, follicular lymphoma; Ibr, ibrutinib; Len, lenalidomide; LDH, lactate dehydrogenase; MCL, mantle cell lymphoma; MZL, marginal zone lymphoma; R, rituxiumab; PD, progressive disease; SD, stable disease; SLL, small lymphocytic lymphoma; ULN, upper limit of normal.

^**a**^ Rituximab dose was 375 mg/m^2^.

As of the clinical data cutoff date of March 6, 2019, 16 patients had continued to receive treatment, 9 patients completed assigned treatment (none received ibrutinib), and 81 patients had discontinued treatment. Additional data describing treatment discontinuation by treatment arm are available in Table S2. The median duration of follow‐up was 23.0 months in Arm A, 23.3 months in Arm B, 14.8 months in Arm C, and 23.3 months in Arm D. During dose‐finding, the median number of durvalumab treatment cycles ranged from 6.5 to 12 (Arm A), 9 to 13 (Arm B), 1 to 9 (Arm C). During dose‐confirmation, the median number of durvalumab treatment cycles ranged from 5 to 13 (Arm B), 2 to 10 (Arm C), and 1.5 to 4 (Arm D). The median number of ibrutinib treatment cycles in Arm B was 5 (MCL) and 22.5 (CLL/SLL), and the median number of bendamustine treatment cycles in Arm C was 2 (DLBCL), 3 (CLL/SLL), and 6 (FL).

### 3.2 Safety

#### 3.2.1 Confirmation of RP2D

Across the clinical program, the RP2D for durvalumab was previously determined to be 1500 mg Q4W in patients weighing more than 30 kg and was not examined in the current trial.^15^ The safety review committee did not confirm the RP2D for rituximab or lenalidomide in Arm A. For Arm B, ibrutinib 420 mg and 560 mg were confirmed as the RP2D for patients with CLL/SLL and MCL, respectively. For Arm C, RP2D was confirmed as rituximab 375 mg/m^2^ with or without bendamustine 70 mg/m^2^; patients with CLL were not included in the dose‐confirmation part in this arm. Neither the nontolerated dose nor the maximum tolerated dose was defined. No dose escalation occurred for patients receiving durvalumab, either as monotherapy or when given in combination with other therapies. In Arm C, patients initially received durvalumab + rituximab, which was subsequently escalated to durvalumab + rituximab + bendamustine 70 mg/m^2^ followed by durvalumab + rituximab + bendamustine 90 mg/m^2^. Bendamustine 90 mg/m^2^ proved intolerable, so durvalumab + rituximab + bendamustine 70 mg/m^2^ was used during dose confirmation.

In Arm A, three of three patients receiving durvalumab in combination with rituximab and lenalidomide 20 mg experienced dose‐limiting thrombocytopenia (grade 4), headache (grade 3), and hepatitis (grade 3); one ** **patient receiving durvalumab in combination with rituximab and lenalidomide 10 mg experienced febrile neutropenia (grade 3), and no DLTs were observed for the three patients receiving durvalumab in combination with lenalidomide 20 mg. In Arm B, no DLTs were observed. In Arm C, one DLT of grade 4 neutropenia was observed in a patient receiving durvalumab in combination with rituximab and bendamustine 90 mg/m^2^.

**FIGURE 2:**
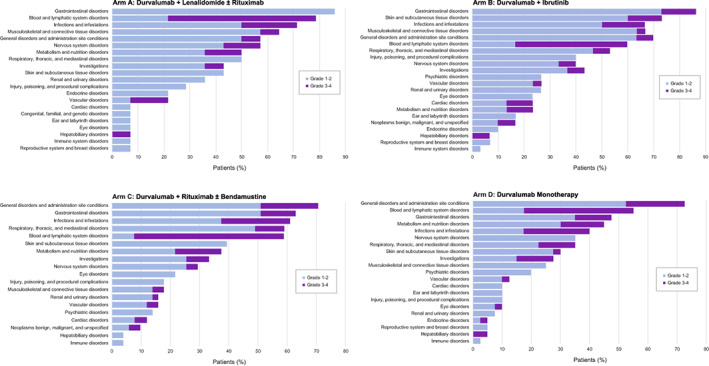
Treatment‐emergent adverse events by treatment arm (safety population)

All but two patients (one in Arm C [DLBCL] and one in Arm D [DLBCL during the durvalumab monotherapy treatment period]) experienced at least one TEAE. The TEAEs are shown by study arm in Figure 2. The most common AEs in Arm A and Arm B were gastrointestinal in nature (Arm A, 85.7%; Arm B, 94.4%) while general disorders and administration site conditions (e.g. pyrexia, fatigue, asthenia, peripheral edema) were most common in Arm C (70.6%) and Arm D (75.0%). Among patients with FL across all study arms, there were six on‐study deaths (two in Arm A, four in Arm D); four were related to disease progression, one to a second primary malignancy (bladder cancer, Arm A), and one from an unknown cause. Among patients with DLBCL across study arms, there were 30 on‐study deaths (two in Arm A, 19 in Arm C, and 9 in Arm D); 27 were related to disease progression, two from unknown causes, and one from respiratory failure. Among patients with MCL, there was one death in Arm B due to a treatment‐related AE (pneumonitis) that was attributed to both durvalumab and ibrutinib and one in Arm D due to disease progression. There were two deaths among patients with CLL/SLL in Arm C, one due to an AE (sepsis) that was unrelated to study treatment and one due to a lung infection that occurred after the treatment period. There were three deaths in patients with HL in Arm D after the treatment period; two patients receiving monotherapy died from unknown causes after the treatment period and one patient receiving combination treatment died from respiratory failure.

### 3.3 Efficacy

Treatment responses are shown for this study in Table 2. During dose finding, patients in Arm A with R/R NHL receiving either durvalumab + lenalidomide 20 mg or durvalumab + rituximab 375 mg/m^2^ and lenalidomide 20 mg had an ORR of 66.7%; those receiving durvalumab + rituximab 375 mg/m^2^ and lenalidomide 10 mg had an ORR of 80%.

**TABLE 2:** Treatment response by treatment arm (efficacy population)

Parameter	Arm A durvalumab combinations			Arm B durvalumab combinations		Arm C durvalumab combinations			Arm D durvalumab monotherapy 1500 mg				
Dose‐finding cohort	Len 20 mg	R^a^ + Len 20 mg	R^a^ + Len 10 mg	Ibr 420 mg	Ibr 560 mg	R^a^	R^a^ + Ben 70 mg	R^a^ + Ben 90 mg	No dose‐finding				
*n*	3	3	5	3	4	3	4	4					
Response, *n* (%)													
CR	1 (33.3)	1 (33.3)	1 (20)	1 (33.3)	0	0	1 (25)	0					
PR	1 (33.3)	1 (33.3)	3 (60)	1 (33.3)	3 (75)	1 (33.3)	1 (25)	0					
SD	1 (33.3)	1 (33.3)	1 (20)	1 (33.3)	1 (25)	1 (33.3)	0	1 (25)					
PD	0	0	0	0	0	1 (33.3)	2 (50)	1 (25)					
NE/ND	0	0	0	0	0	0	0	2 (50)					
ORR	2 (66.7)	2 (66.7)	4 (80)	2 (66.7)	3 (75)	1 (33.3)	2 (50)	0					
95% CI	9.4, 99.2	9.4, 99.2	28.4, 99.5	9.4, 99.2	19.4, 99.4	0.8, 90.6	6.8, 93.2	NE, NE					

Dose‐confirmation cohort	No dose‐confirmation			Ibr 420 mg	Ibr 560 mg	R^a^ + Ben 70 mg			Durvalumab monotherapy 1500 mg				
				CLL/SLL	MCL	FL	DLBCL	CLL/SLL	FL	DLBCL	CLL/SLL	MCL	HL
*n*				9	10	9	10	4	5	10	2	5	5
Response, *n* (%)													
CR				0	3 (30)	4 (44.4)	1 (10)	1 (25)	0	0	0	0	0
PR				9 (100)	4 (40)	4 (44.4)	2 (20)	1 (25)	0	0	0	0	1 (20)
SD				0	0	0	1 (10)	1 (25)	1 (20)	2 (20)	0	2 (40)	1 (20)
PD				0	2 (20)	0	5 (50)	1 (25)	3 (60)	6 (60)	2 (100)	3 (60)	3 (60)
NE/ND				0	0	1 (10)	0	0	0	0	0	0	0
ORR				9 (100)	7 (70)	8 (88.9)	3 (30)	2 (50)	0	0	0	0	1 (20)
95% CI				66.4, 100.0	34.8, 93.3	51.8, 99.7	6.7, 65.2	6.8, 93.2	NE, NE	NE, NE	NE, NE	NE, NE	0.5, 71.6

Abbreviations: Ben, bendamustine; CI, confidence interval; CLL, chronic lymphocytic leukemia; CR, complete response; DLBCL, diffuse large B cell lymphoma; FL, follicular lymphoma; HL, Hodgkin lymphoma; Ibr, ibrutinib; Len, lenalidomide; MCL, mantle cell lymphoma; ND, not done; NE, not estimable; ORR, overall response rate; PD, progressive disease; PR, partial response; R, rituximab; SD, stable disease; SLL, small lymphocytic lymphoma.

^**a**^ Rituximab dose was 375 mg/m^2^.

Similar responses were seen in patients with R/R NHL or R/R CLL from Arm B (durvalumab + ibrutinib 420 mg, 66.7%; durvalumab + ibrutinib 560 mg, 75%). The worst response seen during the dose‐finding part in these patients was stable disease; no patient had progressive disease. During the dose‐confirmation part, patients in Arm B with CLL/SLL who received durvalumab + ibrutinib 420 mg had an ORR of 100% and patients in Arm B with MCL who received durvalumab + ibrutinib 560 mg had an ORR of 70%. For patients in Arms A, B, and C with FL (*n* = 23), the ORR was 59% (27% CR); the ORR among patients with DLBCL (*n* = 37) was 18% (8% achieved CR).

**FIGURE 3:**
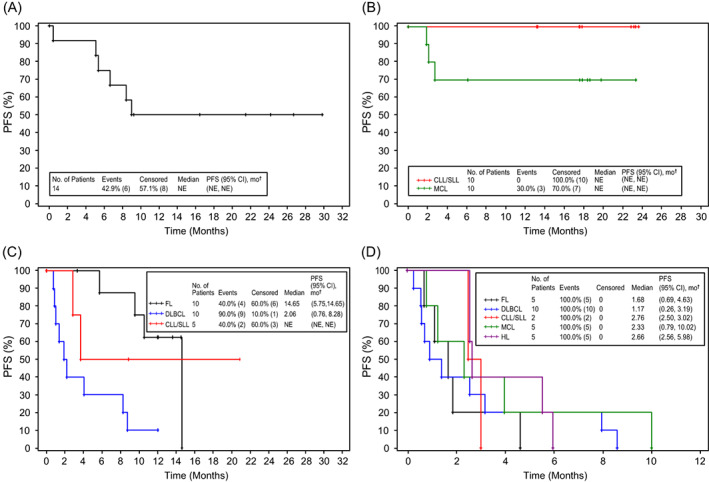
Kaplan–Meier analysis of progression‐free survival for the safety population. Panel A shows estimated progression‐free survival for the Arm A dose‐finding cohort, all histologies. Of the 14 patients in the cohort, 6 (43%) had an event. Panel B shows estimated progression‐free survival for the Arm B dose‐confirmation cohort for patients with CLL/SLL and MCL histologies. Of the 20 patients in the cohort, 3 (15%) had an event, all patients with MCL histology. Panel C shows estimated progression‐free survival for the Arm C dose‐confirmation cohort for patients with FL, DLBCL, and CLL/SLL histologies. Of the 25 patients in the cohort, 15 (60%) had an event, 4/10 (40%) with FL, 9/10 (90%) with DLBCL, and 2/5 (40%) with CLL/SLL. Panel D shows estimated progression‐free survival for the Arm D dose‐confirmation cohort for patients with FL, DLBCL, CLL/SLL, MCL, and HL histologies. Of the 27 patients in the cohort, all had an event. ^†^Brookmeyer Crowley two‐sided 95% CI of the median based on log–log transformation. (A) Arm A: Durvalumab + Lenalidomide ± Rituximab (dose finding). (B) Arm B: Durvalumab + Ibrutinib (dose confirmation). (C) Arm C: Durvalumab + Rituximab ± Bendamustine (dose confirmation). (D) Arm D: Durvalumab Monotherapy (dose confirmation). CI, confidence interval; CLL, chronic lymphocytic leukemia; DLBCL, diffuse large B cell lymphoma; FL, follicular lymphoma; HL, Hodgkin lymphoma; MCL, mantle cell lymphoma; SLL, small lymphocytic lymphoma.

**FIGURE 4:**
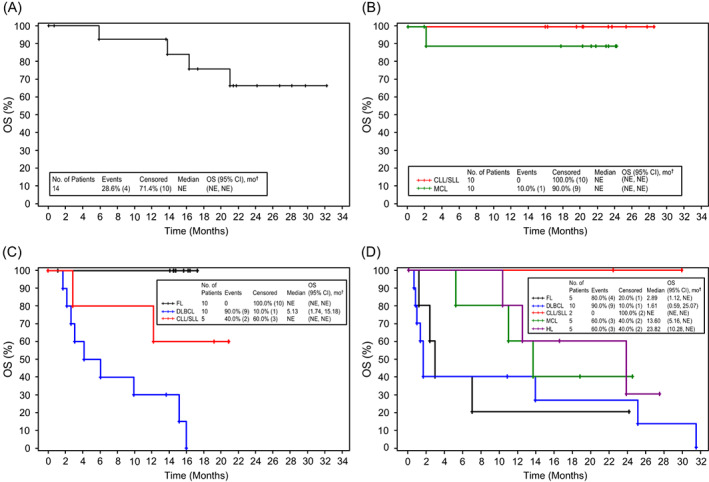
Kaplan–Meier analysis of overall survival for the safety population. Panel A shows Kaplan–Meier overall survival estimates for the Arm A dose‐finding cohort, all histologies. Of the 14 patients in the cohort, 4 (29%) had died. Panel B shows Kaplan–Meier overall survival estimates for the Arm B dose‐confirmation cohort for patients with CLL/SLL and MCL histologies. Of the 20 patients in the cohort, 1 (10%) patient with MCL had died. Panel C shows overall survival estimates for the Arm C dose‐confirmation cohort for patients with FL, DLBCL, and CLL/SLL histologies. Of the 25 patients in the cohort, 11 (44%) had died, 9/10 (90%) with DLBCL and 2/5 (40%) with CLL/SLL. Panel D shows overall survival estimates for the Arm D dose‐confirmation cohort for patients with FL, DLBCL, CLL/SLL, MCL, and HL histologies. Of the 27 patients in the cohort, 19 (70%) had died, 4/5 (80%) with FL, 9/10 (90%) with DLBCL, 3/5 (60%) with MCL, and 3/5 (60%) with HL. ^†^Brookmeyer Crowley two‐sided 95% CI of the median based on log–log transformation. (A) Arm A: Durvalumab + Lenalidomide ± Rituximab (dose finding). (B) Arm B: Durvalumab + Ibrutinib (dose confirmation). (C) Arm C: Durvalumab + Rituximab ± Bendamustine (dose confirmation). (D) Arm D: Durvalumab Monotherapy (dose confirmation). CI, confidence interval; CLL, chronic lymphocytic leukemia; DLBCL, diffuse large B cell lymphoma; FL, follicular lymphoma; HL, Hodgkin lymphoma; MCL, mantle cell lymphoma; SLL, small lymphocytic lymphoma.

Median TTR, DoR, PFS, and OS this study are provided in Table S3. Median DoR was not estimable for most patient cohorts; the exception was patients with DLBCL from the dose‐confirmation part of Arm C receiving durvalumab + rituximab 375 mg/m^2^ and bendamustine (ORR = 24.1% [95% CI: 9.1, 26.1]). Kaplan–Meier analyses of PFS and OS in each arm are shown in Figures 3 and 4, respectively. Median PFS for patients with FL was 9.6 months (95% CI: 4.6, not estimable [NE]) and median OS was not mature. Patients with DLBCL had a median PFS of 2.5 months (95% CI: 1.3, 5.4); median OS was 7.9 months (95% CI: 2.2, 15.3). In the dose‐confirmation of Arm D, median PFS for patients with CLL/SLL was 2.8 months (95% CI: 2.5, 3.0); median PFS and OS of patients with MCL was 2.3 months (95% CI: 0.8, 10.0) and 13.6 months (95% CI: 5.2, NE), respectively; patients with HL had a median PFS and OS of 2.7 months (95% CI: 2.6, 6.0) and 23.8 months (95% CI: 10.3, NE).

### 3.4 Exploratory biomarker analysis

**FIGURE 5:**
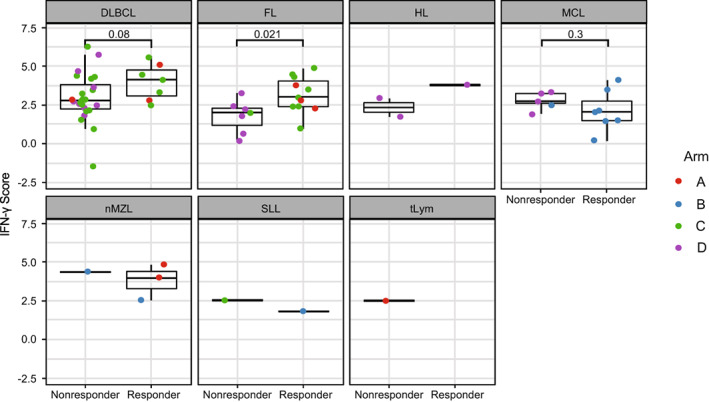
IFN‐γ RNA‐seq score split by best response by histology. The IFN‐γ signature score is shown for all non‐CLL patients and is split into panels by histology. The points are colored based on the Arm, and a *t*‐test was performed comparing the patients with a best overall response of response vs those with no response. CLL, chronic lymphocytic leukemia; DLBCL, diffuse large B cell lymphoma; FL, follicular lymphoma; HL, Hodgkin lymphoma; IFN, interferon; MCL, mantle cell lymphoma; nMZL, nodal marginal zone lymphoma; SLL, small lymphocytic lymphoma.

**FIGURE 6:**
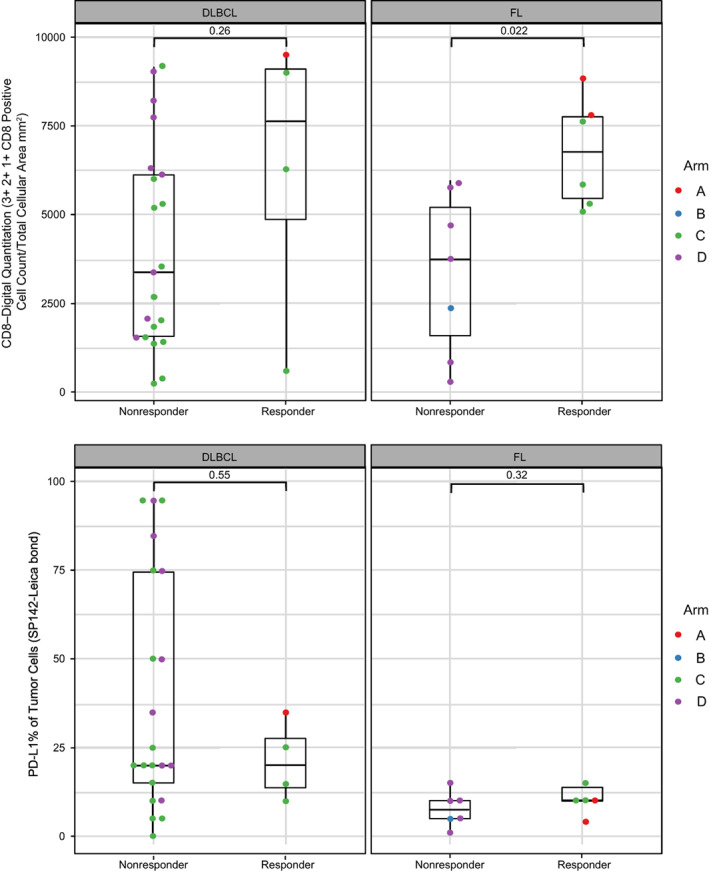
IHC results split by best response for (A) CD8, and (B) PD‐L1. IHC, immunohistochemistry; PD‐L1, programmed death ligand 1

The biomarker analysis is represented in Figures 5 and 6. Notably, in our study, patient samples from patients with FL who responded to therapy across all of the arms of the study exhibited a slightly higher IFN‐γ signature score than nonresponders (*p* = .02), while patients with DLBCL trended in the same direction (*p* = .08; Figure 5).

We evaluated PD‐L1 and CD8 expression in baseline tumor biopsies by IHC and correlated expression with response (responders vs. nonresponders). PD‐L1 did not trend differently in responders compared with nonresponders (Figure 6) in either FL or DLBCL. Our analysis shows that responders had a slightly higher CD8 density at baseline in FL (*p* = .022), indicating a significant correlation between CD8 density and response in FL. There was no CD8 density difference in DLBCL, although there were only four DLBCL responder samples for IHC. CD8 density was not significant in other histologies.

## 4 DISCUSSION

Many existing studies have reported additional benefits of the combination therapies included in the current report in patients with various hematologic malignancies. In the phase 2 PCYC‐1104 study (NCT01236391),^26^ 111 patients with R/R MCL received ibrutinib 560 mg daily and had an ORR of 68% (*n* = 75), with a complete response rate of 21% and a partial response rate of 47%. With a median follow‐up of 15.3 months, median PFS was 13.9 months and median overall survival was not reached. RESONATE was a multicenter, open‐label, phase 3 study (NCT01578707) in which 391 patients with R/R CLL or SLL were randomly assigned to receive daily ibrutinib or the anti‐CD20 antibody ofatumumab. In this study, the investigator‐assessed ORR was significantly greater in the ibrutinib group than in the ofatumumab group (85% vs. 23%, *p* < .001).^27^ Although caution should be made when making cross‐study comparisons, especially due to the small numbers in our study, during the dose‐confirmation part of FUSION NHL 001, patients treated with durvalumab plus ibrutinib 560 mg (*n* = 10) had and ORR of 70%, of which 30% were complete and 40% were partial responses; neither median PFS or median overall survival was reached. The phase 3 AUGMENT study found that patients with R/R FL or marginal zone lymphoma (*N* = 358) treated with lenalidomide plus rituximab had improved ORR versus those receiving placebo plus rituximab (78% vs. 53%, *p* < .0001).^28^ Although the dose‐finding part of FUSION NHL 001 also included patients with DLBCL (*n* = 4), those treated with lenalidomide (either 10 or 20 mg) plus rituximab (*n* = 8) had an ORR of 75%. While most studies of rituximab plus bendamustine focus on treatment‐naive disease, in a retrospective multicenter analysis of 55 Italian patients with R/R DLBCL the ORR after rituximab plus bendamustine was 50%, including a 28% complete remission rate; median overall survival was 10.8 months.^29^ During the dose‐confirmation part of FUSION NHL 001, patients with DLBCL (*n* = 10) treated with rituximab plus bendamustine had an ORR of 30%; 10% achieved a complete response.

The present study investigated the safety and efficacy of durvalumab in various combinations with standard therapy and as monotherapy for relapsed/refractory lymphomas and CLL. The TEAE profile was consistent with the respective safety profiles for the study treatments, and no new safety concerns were identified. Almost all patients experienced a TEAE during FUSION NHL 001, perhaps indicating the difficulty of combining durvalumab with full doses of other therapeutic agents. A more detailed evaluation of optimum dosing may be warranted. Despite a strong rationale for the combination of ibrutinib with PD‐1/PD‐L1‐targeted therapy and preclinical evidence in murine lymphoma models for synergistic antitumor activity with ibrutinib plus durvalumab,^3^ the highest responses seen in our study during dose confirmation (90%; Arm B, durvalumab 1500 mg + ibrutinib 420 mg) and dose finding (75%; Arm B, durvalumab 1500 mg + ibrutinib 560 mg) were similar to or lower than previous reports of responses to ibrutinib monotherapy among patients with relapsed/refractory CLL.^30^

Biomarker analysis showed that slightly higher IFN‐γ signature scores were correlated with response to therapy in patients with FL who responded to therapy across all of the arms of the study. This may suggest that the use of an IFN‐γ gene signature may serve as a biomarker by which to enrich for patients that may be more responsive to anti‐PD‐L1‐based therapy and will require further investigation. These data further indicate the role of the IFN‐γ pathway as an important component of the tumor response to durvalumab treatment, particularly in patients with FL.

Limitations to this study included the discontinuation of enrollment in one study arm due to a decision of the FDA, based on the risks of the anti‐PD‐1 antibody pembrolizumab in combination with immunomodulatory drugs in patients with multiple myeloma, to place a partial clinical hold on five clinical trials and place a full clinical hold on another trial that was evaluating durvalumab in combination with other agents for hematologic malignancies. Based on this FDA decision, the sponsor did not open the planned dose‐expansion part of this study. In addition, the nonrandomized nature of this phase 1/2 trial made definitive conclusions regarding the utility of durvalumab difficult to assess.

## 5 CONCLUSIONS

Based on initial studies that suggested promising antitumor activity with PD‐1/PD‐L1 blockade, we undertook studies to evaluate the safety and activity of anti‐PD‐1/PD‐L1‐based combinations. Despite existing rationale, durvalumab alone and durvalumab‐based combinations did not provide additional benefit while being associated with the toxicity of PD‐L1 blockade. A randomized clinical trial is needed to clarify the role of checkpoint inhibitors in this therapeutic space. In addition, the high rate of TEAEs during this study may reflect the difficulty in combining durvalumab with full doses of other agents. However, the use of an IFN‐γ gene signature may serve as a biomarker by which to enrich for relapsed or refractory patients with enhanced response to anti‐PD‐L1‐based therapy and will require further investigation.

## AUTHOR CONTRIBUTIONS

*Conceptualization*, N.K., T.W., M.C.; *Methodology*, N.K., T.W., M.C.; *Software*, N.K., N.R., L.T., J.D., T.W., R.D., B.F., J.M., M.C.; *Validation*, N.K., N.R., L.T., J.D., T.W., R.D., B.F., J.M., M.C.; *Investigation*, C.C., N.K., N.R., L.T., J.D., T.W., R.D., J.R., J.M., B.F., M.C.; *Formal Analysis*, N.K., N.R., L.T., J.D., T.W., R.D., B.F., J.M., M.C.; *Resources*, N.K., T.W., M.C.; *Data Curation*, C.C., N.K., N.R., L.T., J.D., T.W., R.D., J.R., J.M., B.F., M.C.; *Writing, Original Draft*, T.W., J.M., B.F.; *Writing, Review & Editing*, All authors; *Visualization*, N/A; *Supervision*, C.C., P.L., J.R., J.M.; *Project Administration*, N/A; *Funding Acquisition*, N/A.

## FUNDING INFORMATION

This trial (NCT02733042) was sponsored by Celgene, a Bristol Myers Squibb company, and supported by AstraZeneca/MedImmune.

## CONFLICT OF INTEREST

Carla Casulo: Research funding: Verastem, Gilead, Genentech, Bristol Myers Squibb. Armando Santoro: Consulting or advisory role: ArQule, Bayer, Bristol Myers Squibb, Eisai, Gilead Sciences, Incyte, MSD, Pfizer, Sanofi, Servier; Speakers' bureau: AbbVie, Amgen, ArQule, AstraZeneca, Bayer, Bristol Myers Squibb, Celgene, Eisai, Gilead Sciences, Lilly, MSD, Novartis, Pfizer, Roche, Sandoz, Servier, Takeda. Guillaume Cartron: Consultant: Roche, Celgene; Honorarium: Sanofi, Jansen, Gilead, Novartis, Celgene, Roche. Kiyoshi Ando: Celgene, Novartis, Kyowa Kirin Co., Ltd, Chugai Pharmaceutical Co., Ltd, Takeda Pharmaceutical Company, Limited. Javier Munoz: Consulting: Pharmacyclics, Bayer, Gilead/Kite Pharma, Pfizer, Janssen, Juno/Celgene, Bristol Myers Squibb, Kyowa, Alexion, Beigene, Fosunkite, Innovent, Seattle Genetics, Beigene; Research funding: Bayer, Gilead/Kite Pharma, Celgene, Merck, Portola, Incyte, Genentech, Pharmacyclics, Seattle Genetics, Janssen, Millennium. Honoraria: Kyowa and Seattle Genetics; Speakers bureau: Gilead/Kite Pharma, Kyowa, Bayer, Pharmacyclics/Janssen, Seattle Genetics, Acrotech/Aurobindo, Beigene, Verastem, AstraZeneca, Celgene/BMS, Genentech/Roche, AbbVie. Steven Le Gouill: Nothing to disclose. Koji Izutsu: Honararia: Celgene, Eisai, Chugai. Simon Rule: Nothing to disclose. Jia Ruan: Research grants: Bristol Myers Squibb, Daiichi Sankyo, AstraZeneca, Pharmacyclics, Seagen; Consultancy: Bristol Myers Squibb, Daiichi Sankyo, Seagen. Luca Arcaini: Advisory honoraria: Roche, Celgene, Janssen‐Cilag, Verastem, Eusa Pharma, Incyte; Research support: Gilead. Pieternella Lugtenburg: Research funding: Takeda, Servier; Honoraria for advisory boards: Takeda, Servier, Genmab, Regeneron, Incyte, Roche, Celgene. Brian Fox, Nurgul Kilavuz, Justine Dell'Aringa, Nils Rettby, Lilia Taningco, Myron Czuczman: Employees of and shareholders of Bristol Myers Squibb. Marie‐Laure Casadebaig, Richard Delarue: Employees of Bristol Myers Squibb and may be shareholders. Thomas Witzig: The Mayo Clinic received research support for this trial. Advisory boards: Celgene (personally uncompensated).

## ETHICS STATEMENT

The study was conducted in compliance with the International Council for Harmonization Good Clinical Practice and in accordance with the general ethical principles outline in the Declaration of Helsinki and applicable national, state, and local laws of the pertinent regulatory authorities. The protocol was approved by an institutional review board/independent ethics committee prior to study commencement.

## Supporting information


**Table S1** Complete Inclusion/Exclusion Criteria Overall and by Treatment Arm
**Table S2.** Subject Disposition by Treatment Arm and Study Part (Safety Population)
**Table S3.** Time‐to‐Event Analyses

## Data Availability

The Bristol Myers Squibb policy on data sharing may be found at https://www.bms.com/researchers-and-partners/independent-research/data-sharing-request-process.html
