# Supplementary material for: Durvalumab as monotherapy and in combination therapy in patients with lymphoma or chronic lymphocytic leukemia: The FUSION NHL 001 trial
Source: Cancer Rep (Hoboken). 2022 Jul 19;6(1):e1662. doi: 10.1002/cnr2.1662 (PMC9875673; doi:10.1002/cnr2.1662)
Supplement: Supplementary file 1 — Table S1 Complete Inclusion/Exclusion Criteria Overall and by Treatment Arm Table S2. Subject Disposition by Treatment Arm and Study Part (Safety Population) Table S3. Time‐to‐Event Analyses [file CNR2-6-e1662-s001.docx]

**Durvalumab as monotherapy and in combination therapy in patients with lymphoma or chronic lymphocytic leukemia: the FUSION NHL 001 trial**

Carla Casulo, MD^1^; Armando Santoro^2^; Guillaume Cartron, MD, PhD^3^; Kiyoshi Ando^4^; Javier Munoz, MD, MS, FACP^5^; Steven Le Gouill, MD, PhD^6^; Koji Izutsu, MD, PhD^7^; Simon Rule^8^; Pieternella Lugtenburg, MD, PhD^9^; Jia Ruan^10^; Luca Arcaini, MD^11^; Marie-Laure Casadebaig, MsC^12^; Brian Fox^12^; Nurgul Kilavuz, MS^12^*; Nils Rettby, MS^12^; Justine Dell’Aringa, BS^12^; Lilia Taningco, BA^12^; Richard Delarue, MD^12*^; Myron Czuczman, MD^12*^; Thomas Witzig, MD^13^

**Affiliations:** ^1^Wilmot Cancer Institute, University of Rochester Medical Center, Rochester, NY, USA; ^2^Humanitas University and Humanitas Clinical and Research Center IRCCS, Rozzano-Milan, Italy; ^3^Centre Hospitalier Universitaire de Montpellier, Montpellier, France; ^4^Tokai University School of Medicine, Isehara, Japan; ^5^Banner MD Anderson Cancer Center, Gilbert, AZ, USA; ^6^Service d’hématologie clinique du CHU de Nantes, INSERM CRCINA Nantes-Angers, NeXT Université de Nantes, Nantes, France; ^7^National Cancer Center Hospital, Tokyo, Japan; ^8^University of Plymouth, Plymouth, UK; ^9^Erasmus MC Cancer Institute, University Medical Center Rotterdam, Rotterdam, The Netherlands; ^10^Weill Cornell Medicine, New York, NY, USA; ^11^Division of Hematology, Fondazione IRCSS Policlinico San Matteo and Department of Molecular Medicine, University of Pavia, Pavia, Italy; ^12^Bristol Myers Squibb, Princeton, NJ, USA;^13^Mayo Clinic, Rochester, MN, USA

*Affiliation at the time of the study.

**Table S1. Complete** **Inclusion/Exclusion Criteria Overall and by Treatment Arm**

| **Inclusion criteria** | **Exclusion criteria** |
| --- | --- |
| **All treatment arms** | |
| 1. Patient was ≥18 years of age and ≤80 years of age at the time of signing the informed consent form (ICF). 2. Exception: At the discretion of the investigator, patients >80 years of age could have been included if their Eastern Cooperative Oncology Group (ECOG) performance status was ≤1; each of their individual organ system scores must have been ≤2 using the Modified Cumulative Illness Rating Scale for comorbidity (Salvi F, et al. *J Am Geriatr Soc*. 2008;56(10)1926-1932; Salvi F, et al. *J Am Geriatr Soc*. 2008;56; Appendix I of the protocol [Appendix 16.1.1]). 3. Patient must have understood and voluntarily signed an ICF prior to any study-related assessments/procedures being conducted. 4. Patient was willing and able to adhere to the study visit schedule and other protocol requirements. 5. Patient had histologically confirmed and documented eligible histologies as listed in Table 3, and Table 4 and Table 5 of the protocol (Appendix 16.1.1) as assessed by the investigator and local pathologist per the 2008 WHO Lymphoma Classification (Swerdlow SH, et al. World Health Organization Classification of Tumours of Haematopoietic and Lymphoid Tissues, IARC Press, Lyon 2008). Eligible subhistologies for the dose-confirmation and/or dose-expansion cohorts:    1. Follicular lymphoma (FL) cohorts: FL Grade 1, 2, and 3a    2. Diffuse large B-cell lymphoma (DLBCL; de novo) cohorts: DLBCL not otherwise specified, T-cell/histiocyte rich large B-cell lymphoma, and FL Grade 3b    3. Mantle cell lymphoma (MCL) cohort: MCL    4. Chronic lymphocytic leukemia (CLL)/small lymphocytic leukemia (SLL) cohorts: high-risk CLL/SLL    5. Hodgkin lymphoma (HL) cohort: cHL, nodular sclerosing cHL, lymphocyte-rich cHL, mixed cellularity cHL, lymphocyte-depleted cHL 6. Patient had been previously treated with at least 1 prior systemic chemotherapy, immunotherapy, or chemoimmunotherapy.    1. Note: Local involved field radiation therapy or antibiotic-based therapy was not deemed as systemic therapy for this study. 7. Patient with high-risk CLL/SLL was defined by the presence of at least 1 of the following factors:    1. Complex karyotype    2. del (17p) abnormality    3. Mutated TP53    4. Ibrutinib or other Bruton’s tyrosine kinase (BTK) inhibitor failure (defined as progression while on ibrutinib treatment [excluding isolated early lymphocytosis]; or an inadequate tumor response which is less than partial response [PR] [Hallek M, et al. *Blood*. 2008;111(12):5446-5456; Cheson BD, et al. *J Clin Oncol*. 2014;32(27):3059-3068]).    5. Relapsed/progressive disease (PD) within 6 months of completing their last therapy which may include investigational drug. 8. Patient was willing and able to undergo biopsy:    1. Patient with lymphoma was willing and able to undergo tumor/lymph node biopsy (incisional/excisional or multiple core needle).       - During the Screening Period       - Any time during Cycle 2 (strongly recommended), and       - At the time of disease progression from patients who had achieved objective response (complete response [CR]/PR) to study treatment.       - Patient with CLL was willing and able to undergo bone marrow biopsy (BMB) during the Screening and Treatment Periods.    2. Material from a fine needle aspiration was not acceptable. 9. Patient who had documented active relapsed or refractory disease requiring therapeutic intervention. 10. Patient who had measurable disease:     1. For patient with lymphoma, bidimensionally measurable disease on cross-sectional imaging by computed tomography (CT) with at least 1 nodal or extranodal lesion ≥2.0 cm in its longest dimension. Note: A previously irradiated lesion was ineligible to be used as a measurable target lesion.     2. For patient with CLL, in need of treatment as defined by International Workshop on CLL (IWCLL) Guidelines for the Diagnosis and Treatment of CLL (Appendix H of the protocol [Appendix 16.1.1]). 11. Patient who had performance status of 0, 1, or 2 on the ECOG scale. 12. Patient who had life expectancy of greater than 6 months. 13. Patient who fulfilled the laboratory requirements in Table 6 of the protocol (Appendix 16.1.1). 14. Female patient of childbearing potential (FCBP) who was sexually active with a male had to:     1. Have 2 negative pregnancy tests as verified by the investigator prior to starting any investigational product (IP) therapy. They must have agreed to ongoing pregnancy testing during the course of the study, and after the last dose of any IP. This applied even if the patient practiced true abstinence from heterosexual contact.     2. Use effective methods (1 highly effective and 1 additional effective [barrier] method) of contraception from 28 days prior to starting durvalumab, and must have agreed to continue using such precautions while taking durvalumab (including dose interruptions) and for 90 days after the last dose of durvalumab. Cessation of contraception after this point should have been discussed with a responsible physician. The following are examples of highly effective and additional effective methods of contraception:  - Highly effective methods (defined as one that results in a low failure rate [ie, less than 1% per year] when used consistently and correctly):   - - - 1. Intrauterine device         2. Hormonal (birth control pills, injections, implants, levonorgestrel-releasing intrauterine system, medroxyprogesterone acetate depot injections, ovulation inhibitory progesterone-only pills [eg, desogestrel])         3. Tubal ligation         4. Partner vasectomy       - Additional effective methods:         1. Male condom         2. Diaphragm         3. Cervical cap   1. Agree to abstain from breastfeeding during study participation and for at least 90 days after the last dose of durvalumab.   2. Refrain from egg cell donation while taking durvalumab and for at least 90 days after the last dose of durvalumab.  1. Male patient who was sexually active with a female partner of childbearing potential had to:    1. Use male condom plus spermicide (even if he had undergone a successful vasectomy) from starting dose of durvalumab (Cycle 1 Day 1) through 90 days after receipt of the last dose of durvalumab. True abstinence was acceptable only when this was in line with the preferred and usual lifestyle of nonsterilized male patient.    2. Refrain from semen or sperm donation while taking durvalumab and for at least 90 days after the last dose of durvalumab. | 1. Patient who had known or suspected central nervous system (CNS) or meningeal involvement by lymphoma. 2. Patient who had other lymphoma histologies which are not listed on Table 3, and Table 4 and Table 5 of the protocol (Appendix 16.1.1; e.g., human immunodeficiency virus [HIV]-associated lymphomas, CNS lymphoma, Waldenstrom’s macroglobulinemia).    1. Patient had blastoid variants of MCL or MCL with blastoid transformation.    2. Dose-confirmation and/or Dose-expansion Parts only:       - Transformed lymphoma or RT       - Diffuse large B-cell lymphoma histology other than: not otherwise specified or T-cell/histiocyte rich 3. Patient who had any histopathologic finding consistent with myelodysplastic syndrome on bone marrow studies. 4. Patient who had any significant medical condition, laboratory abnormality, or psychiatric illness that would have prevented the patient from participating in the study. 5. Patient who had any condition including the presence of laboratory abnormalities, which placed the patient at unacceptable risk if he/she were to participate in the study. 6. Patient who had any condition that confounded the ability to interpret data from the study. 7. Patient who had any uncontrolled intercurrent illness including, but not limited to, ongoing or active infection, current pneumonitis, symptomatic congestive heart failure, uncontrolled hypertension, unstable angina pectoris, cardiac arrhythmia, interstitial lung disease, or psychiatric illness/social situations that would have limited compliance with study requirement, substantially increased risk of incurring adverse events (AEs) from durvalumab and/or other investigational treatment regimens, or compromised the ability of the patient to give written informed consent. 8. Patient who was concurrently enrolled in another clinical study, unless in a Follow-up Period or it was an observational study. 9. Patient who had any concurrent chemotherapy, immunotherapy, biologic, or hormonal therapy for cancer treatment. Note: Concurrent use of hormones for noncancer-related conditions (e.g., insulin for diabetes and hormone replacement therapy) was permitted. 10. Patient who had received:     1. Any systemic antilymphoma/leukemia therapy, or hematopoietic growth factors, blood or platelets transfusions within 14 days prior to the first dose of IP (i.e., Cycle 1 Day 1) and/or     2. Any radioimmunotherapy within 3 months prior to the first dose of IP (i.e., Cycle 1 Day 1).   Exception: The use of hematopoietic growth factors or blood product transfusional support for patients with extensive marrow involvement by lymphoma or CLL could be allowed during the Screening Period after consultation with the sponsor’s medical monitor in the dose-confirmation and expansion cohorts only (see Table 6 of the protocol [Appendix 16.1.1]).   1. Patient who had unresolved toxicities from prior anticancer therapy, defined as having not resolved to National Cancer Institute Common Terminology Criteria for AEs (NCI CTCAE) Version 4.03 ≤Grade 1 with the exception of alopecia and laboratory values listed per the exclusion criteria. Patients with irreversible toxicity that was not reasonably expected to be exacerbated by durvalumab or other investigational treatments could be included (e.g., hearing loss) after consultation with the sponsor’s medical monitor. 2. Patient who received any prior monoclonal antibody (mAb) against programmed cell death 1 (PD-1) or programmed death ligand 1 (PD-L1) and/or any prior:    1. Arm A only: drugs with immunomodulatory and other properties (e.g., lenalidomide, thalidomide)    2. Arm B only: ibrutinib or other BTK inhibitor    3. Arm C only: bendamustine (except dose level 1) 3. Patient who had history of organ transplant or allogeneic hematopoietic stem cell transplantation. 4. Patient who had taken corticosteroids during the last week prior to the first dose of IP (i.e., Cycle 1 Day 1), unless administered at a dose equivalent to ≤10 mg/day prednisone.   Exception: For patients with bulky disease, systemic symptoms, compressive disease, or rapidly progressing adenopathies, prephase treatment with 1 mg/kg/day prednisone, or equivalent, for a maximum of 7 days was permitted prior to Cycle 1 Day 1, at the discretion of the investigator. A washout period did not apply.   1. Patient who had received live, attenuated vaccine within 30 days prior to the first dose of durvalumab (Note: Patients, if enrolled, did not receive live vaccine during the study and for 12 months after last dose of rituximab or until recovery of B-cells and for 120 days after the last dose of durvalumab, whichever was longer). 2. Patient who had undergone major surgical procedure (as defined by the investigator) within 28 days prior to the first dose of the IP (i.e., Cycle 1 Day 1) or was still recovering from prior surgery. 3. Patient who had active documented autoimmune disease (including, but not limited to, inflammatory bowel disease, celiac disease, Wegener syndrome, hemolytic anemia, or immune thrombocytopenic purpura) prior to first dose of durvalumab.   Exception: Type 1 diabetes mellitus and hypothyroidism which were well-controlled.   1. Patient who had history of primary immunodeficiency or tuberculosis. 2. Patient who had known seropositivity for or active infection for HIV or hepatitis C virus. 3. Patient who was seropositive for or active viral infection with hepatitis B virus (HBV) 4. Hepatitis B virus surface antigen (HBsAg) positive 5. Hepatitis B virus surface antigen negative, HBV core antibody (anti-HBc) positive, and detectable viral DNA 6. Note: A patient who was seropositive for anti-hepatitis B surface antibody (HBs) because of prior exposure or vaccination (anti-HBc and HBsAg negative) were eligible. In this case, viral DNA did not need to be tested. 7. Female patient who was pregnant, breastfeeding, or intended to become pregnant during the participation in the study. 8. Patient who had other invasive malignancy within 2 years prior to signing the ICF except for noninvasive malignancies such as cervical carcinoma in situ, nonmelanomatous carcinoma of the skin, ductal carcinoma in situ of the breast, or incidental histologic finding of prostate cancer (T1a or T1b using the tumor, nodes, metastasis [TNM] clinical staging system) that had been surgically cured.    1. Arm A only: Patient who had history of other malignancies, unless the patient had been free of the disease for ≥5 years prior to signing the ICF. Exceptions: History of previously treated basal cell carcinoma of the skin, squamous cell carcinoma of the skin and related localized nonmelanoma skin cancer, carcinoma in situ of the cervix, carcinoma in situ of breast, incidental histologic finding of prostate cancer (T1a or T1b using the TNM clinical staging system). 9. Patient who had known allergy or hypersensitivity to the active substance or any of the excipients, or to other humanized mAbs. |

| **Arm A only** | |
| --- | --- |
| Female patient of childbearing potential had to:   1. Have 2 negative pregnancy tests as verified by the investigator prior to starting any IP therapy (i.e., durvalumab, lenalidomide and rituximab). They must have agreed to ongoing pregnancy testing during the course of the study, and after last dose of any IP. This applied even if the patient practiced true abstinence from heterosexual contact. 2. Either commit to true abstinence from heterosexual contact (which must have been reviewed on a monthly basis) or agreed to use, and been able to comply with, effective (1 highly effective and 1 additional effective method) contraception without interruption, 28 days prior to starting any IP, during the IP therapy (including dose interruptions), and for 12 months after the last dose of rituximab, 90 days after the last dose of durvalumab, or 28 days after the last dose of lenalidomide, whichever was longer. 3. Agree to abstain from breastfeeding during study participation and for at least 28 days after the last dose of lenalidomide or 12 months after the last dose of rituximab, whichever was longer. 4. Refrain from egg cell donation while taking durvalumab and for 90 days after the last dose of durvalumab.   Male patient had to:   1. Practice true abstinence or agree to use a condom during sexual contact with a pregnant female or an FCBP while participating in the study, during dose interruptions and for at least 28 days after the last dose of lenalidomide, or for at least 90 days after the last dose of durvalumab, even if he had undergone a successful vasectomy, whichever was longer. 2. Agree to not donate semen or sperm during the IP therapy and for 28 days after the last dose of lenalidomide or 90 days after the last dose of durvalumab, whichever was longer.   All patients had to:   1. Have an understanding that lenalidomide could have a potential teratogenic risk. 2. Agree to abstain from donating blood while taking lenalidomide therapy and for 28 days after the last dose of lenalidomide therapy or 90 days after the last dose of durvalumab, whichever was longer. 3. Agree not to share lenalidomide with another person. 4. Agree to be counseled about pregnancy precautions and risk of fetal exposure. | 1. Patients with CLL or SLL. 2. Patient who had peripheral neuropathy Grade 3 or 4. 3. Patient who was at risk for a thromboembolic event and was not willing to take prophylactic treatment.   **A and C only:**   1. Patient who did not have CD20 positive lymphoma or CLL. 2. Patient who had hypersensitivity to rituximab. |

| **Arm B only** | |
| --- | --- |
| Female patient of childbearing potential had to:   1. Have 2 negative pregnancy tests as verified by the investigator prior to starting any IP therapy (i.e., durvalumab and ibrutinib). They must have agreed to ongoing pregnancy testing during the course of the study, and after last dose of any IP. This applied even if the patient practiced true abstinence from heterosexual contact. 2. Either commit to true abstinence from heterosexual contact (which must have been reviewed on a monthly basis) or agreed to use, and been able to comply with, effective (1 highly effective and 1 additional effective method) contraception without interruption, 28 days prior to starting IP therapy, during the IP therapy (including dose interruptions) and for 90 days after the last dose of IP. 3. Agree to abstain from breastfeeding during study participation and for at least 90 days after last dose of IP therapy. 4. Refrain from egg cell donation while taking durvalumab and for 90 days after the last dose of durvalumab.   Male patient had to:   1. Practice true abstinence or agree to use a condom during sexual contact with a pregnant female or an FCBP while participating in the study, during dose interruptions and for at least 90 days following the last dose of IP, even if he had undergone a successful vasectomy, whichever was longer. 2. Agree to not donate semen or sperm during the IP therapy and for 28 days after the last dose of ibrutinib or 90 days after the last dose of durvalumab, whichever was longer. | 1. Patient who had transfusion-dependent thrombocytopenia or a history of bleeding disorders or clinical conditions (e.g., gastrointestinal bleeding or constitutional disorders) that may increase the risk of life-threatening bleeding when thrombocytopenic. 2. Patient who had history of stroke or intracranial hemorrhage within 6 months prior to signing the ICF. 3. Patient who received medications that are strong inhibitors or inducers of cytochrome P450 (CYP)3A (e.g., itraconazole, ketoconazole, clarithromycin, ritonavir, phenytoin, pentobarbital, and rifampin) and could not change. 4. Patient who had received concomitant anticoagulation with warfarin or other vitamin K antagonists within 7 days prior to signing the ICF and could not change. The use of other anticoagulants (e.g., heparins) and antiplatelet agents was allowed per investigator’s discretion. Investigator questions regarding this were addressed to the sponsor’s medical monitor or the study country principal investigators. |

| **Arm C only** | |
| --- | --- |
| Female patient of childbearing potential had to:   1. Have 2 negative pregnancy tests as verified by the investigator prior to starting any IP therapy (i.e., durvalumab, bendamustine and rituximab). They must have agreed to ongoing pregnancy testing during the course of the study, and after last dose of any IP. This applied even if the patient practiced true abstinence from heterosexual contact. 2. Either commit to true abstinence from heterosexual contact (which must have been reviewed on a monthly basis) or agreed to use, and been able to comply with, effective (1 highly effective and 1 additional effective method) contraception without interruption, 28 days prior to starting IP therapy, during the IP therapy (including dose interruptions) and for 12 months after the last dose of rituximab or for 90 days after the last dose of the other IP (ie, bendamustine and/or durvalumab) dose, whichever was longer. 3. Agree to abstain from breastfeeding during study participation and for at least 12 months after the last dose of rituximab. 4. Refrain from egg cell donation while taking durvalumab and for 90 days after the last dose of durvalumab.   Male patient had to:   1. Practice true abstinence or agree to use a condom during sexual contact with a pregnant female patient or an FCBP while participating in the study, during dose interruptions and for at least 90 days after the last dose of durvalumab or 6 months after the last dose of bendamustine, even if he had undergone a successful vasectomy, whichever was longer. 2. Agree to not donate semen or sperm during the IP therapy and for at least 90 days after the last dose of durvalumab or 6 months after the last dose of bendamustine, whichever was longer. | 1. Patient who should have concurrently used allopurinol, e.g., because of gout, and was unwilling to switch to another equivalent medication. (Patients with gout were advised to switch to another antigout medication, because of the risk of Stevens-Johnson syndrome observed in patients using bendamustine and allopurinol.)   **A and C only:**   1. Patient who did not have CD20 positive lymphoma or CLL. 2. Patient who had hypersensitivity to rituximab. |

**Table S2. Patient Disposition by Treatment Arm and Study Part (Safety Population)**

|  | **Treatment ongoing** | **Treatment completed** | **Treatment discontinued** |
| --- | --- | --- | --- |
| Arm A | 0 | 2 | 12 |
| Arm B (dose-finding) | 3 | 0 | 4 |
| Arm B (dose-confirmation) | 12 | 0 | 8 |
| Arm C (dose-finding) | 0 | 1 | 12 |
| Arm C (dose-confirmation) | 0 | 6 | 19 |
| Arm D (dose-confirmation) | 1 | 0 | 21 |

Data cutoff date: March 6, 2019

**Table S3. Time-to-Event Analyses**

| **Parameter** | **Arm A**  **Durvalumab combinations** | | | **Arm B**  **Durvalumab combinations** | | **Arm C**  **Durvalumab combinations** | | | | | **Arm D**  **Durvalumab monotherapy 1500 mg** | | | | |
| --- | --- | --- | --- | --- | --- | --- | --- | --- | --- | --- | --- | --- | --- | --- | --- |
| **Dose-finding cohort** | **Len 20 mg** | **R^†^ + Len 20 mg** | **R^†^ + Len 10 mg** | **Ibr**  **420 mg** | **Ibr**  **560 mg** | **R^†^** | **Ben 70 mg** | | **R^†^+ Ben 70 mg** | **R^†^ + Ben 90 mg** | **No dose-finding** | | | | |
| n (efficacy evaluable) | 3 | 3 | 5 | 3 | 4 | 3 | 0 | | 4 | 4 |  |  |  |  |  |
| Median TTR, weeks (range) | 70.9 (12.1–129.6) | 12.6 (12.1–13.1) | 18.2 (11.3–36.1) | 11.9 (11.4–12.3) | 13.4 (12.4–52.9) | 13.0  (13.0–13.0) | – | | 13.1 (12.1–14.1) | – |  |  |  |  |  |
| Median DoR, weeks (95% CI) | 10.1 (NE, NE) | NE (NE, NE) | NE (NE, NE) | NE (NE, NE) | NE (NE, NE) | 29.3 (NE, NE) | – | | NE (NE, NE) | – |  |  |  |  |  |
| n (safety) | 3 | 3 | 8 | 3 | 4 | 3 | 1 | | 4 | 5 |  |  |  |  |  |
| Median PFS, months (95% CI) | 8.4 (5.1, NE) | NE (NE, NE) | NE (NE, NE) | NE (NE, NE) | 28.7 (4.5, NE) | 9.7 (1.6, 12.7) | 1.3 (NE, NE) | | 3.8 (1.3, NE) | 2.5 (0.5, 5.9) |  |  |  |  |  |
| Median OS, months (95% CI) | NE (NE, NE) | NE (NE, NE) | NE (NE, NE) | NE (NE, NE) | NE (NE, NE) | 24.3 (17.1, 31.6) | 1.3 (NE, NE) | | 11.6 (2.0, NE) | 3.1 (1.2, 17.0) |  |  |  |  |  |
| **Dose-confirmation cohort** |  | | | **Ibr**  **420 mg** | **Ibr  560 mg** | **R**^†^ **+ Ben  70 mg** | | | | | **Durvalumab monotherapy  1500 mg** | | | | |
|  |  |  |  | **CLL/**  **SLL** | **MCL** | **FL** | | **DLBCL** | | **CLL/**  **SLL** | **FL** | **DLBCL** | **CLL/**  **SLL** | **MCL** | **HL** |
| n (efficacy evaluable) |  |  |  | 9 | 10 | 9 | | 10 | | 4 | 5 | 10 | 2 | 5 | 5 |
| Median TTR, months (range) |  |  |  | 12.1 (10.9–72.9) | 12.1 (6.6–26.4) | 12.4 (10.3–15.3) | | 12.0 (8.7–12.1) | | 12.1 (12.1–12.1) | – | – | – | – | 13.1 (13.1–13.1) |
| Median DoR, weeks (95% CI) |  |  |  | NE (NE, NE) | NE (NE, NE) | NE (NE, NE) | | 24.1 (9.1, 26.1) | | NE (NE, NE) | – | – | – | – | 11.1 (NE, NE) |
| n (safety) |  |  |  | 10 | 10 | 10 | | 10 | | 5 | 5 | 10 | 2 | 5 | 5 |
| Median PFS, months (95% CI) |  |  |  | NE (NE, NE) | NE (NE, NE) | 14.7 (5.8, 14.7) | | 2.1 (0.8, 8.3) | | NE (NE, NE) | 1.7 (0.7, 4.6) | 1.2 (0.3, 3.2) | 2.8 (2.5, 3.0) | 2.3 (0.8, 10.0) | 2.7 (2.6, 6.0) |
| Median OS, months (95% CI) |  |  |  | NE (NE, NE) | NE (NE, NE) | NE (NE, NE) | | 5.1 (1.7, 15.2) | | NE (NE, NE) | 2.9 (1.1, NE) | 1.6 (0.6, 25.1) | NE (NE, NE) | 13.6 (5.2, NE) | 23.8 (10.3, NE) |

Data cutoff date: March 6, 2019.

^†^Rituximab dose was 375 mg/m^2^.

Ben, bendamustine; CI, confidence interval; CLL, chronic lymphocytic leukemia; DLBCL, diffuse large B cell lymphoma; DoR, duration of response; FL, follicular lymphoma; HL, Hodgkin lymphoma; Ibr, ibrutinib; Len, lenalidomide; MCL, mantle cell lymphoma; ND, not done; NE, not estimable; OS, overall survival; PFS, progression-free survival; R, rituximab; SLL, small lymphocytic leukemia.
